# Determinants of male involvement in the prevention of mother-to-child transmission of HIV programme in Eastern Uganda: a cross-sectional survey

**DOI:** 10.1186/1742-4755-7-12

**Published:** 2010-06-23

**Authors:** Robert Byamugisha, James K Tumwine, Nulu Semiyaga, Thorkild Tylleskär

**Affiliations:** 1 Mbale Regional Referral Hospital, Department of Obstetrics and Gynaecology, PO Box 921, Mbale, Uganda; 2 Department of Paediatrics and Child Health, School of Medicine, Makerere University College of Health Sciences, PO Box 7072, Kampala, Uganda; 3 Infectious Diseases Institute Ltd, Partners in Prevention, PO Box 10314, Kampala, Uganda; 4 Centre for International Health, University of Bergen, Arstadveien 21, N-5009 Bergen, Norway

## Abstract

**Background:**

Mother-to-child transmission of HIV (MTCT) accounts for over 95% of all paediatric HIV infections worldwide. Several studies have shown that male participation in the antenatal care of their spouses together with couple counselling and testing for HIV, increases use of the interventions for HIV prevention. The prevention programme of MTCT (PMTCT) was launched in Uganda in 2000 and Mbale in 2002. Less than 10% of the pregnant women accepted antenatal HIV testing at Mbale Regional Referral Hospital in 2003; couple counselling and testing for HIV was low. Therefore, we conducted the study to determine the level of male involvement and identify its determinants in the PMTCT programme.

**Methods:**

A cross-sectional survey of 388 men aged 18 years or more, whose spouses were attending antenatal care at Mbale Regional Referral Hospital, was conducted in Mbale district, Eastern Uganda. A male involvement index was constructed based on 6 questions. The survey was complemented by eight focus group discussions and five in-depth interviews.

**Results:**

The respondents had a median age of 32 years (inter-quartile range, IQR: 28-37). The majority (74%) had a low male involvement index and only 5% of men accompanied their spouses to the antenatal clinic. Men who had attained secondary education were more likely to have a high male involvement index (OR: 1.9, 95% CI: 1.1-3.3) than those who had primary or no formal education. The respondents, whose occupation was driver (OR: 0.3, 95% CI: 0.1-0.7) or those who had fear of disclosure of their HIV sero-status results to their spouses (OR: 0.4, 95% CI: 0.2-0.8), were less likely to have a high male involvement index. Barriers to male involvement in the PMTCT programme were related to both the poor health system, to socio-economic factors and to cultural beliefs.

**Conclusions:**

Structural and cultural barriers to men's involvement in the PMTCT programme in Mbale district were complex and interrelated. Community sensitization of men about the benefits of antenatal care and PMTCT and improving client-friendliness in the clinics needs to be prioritised in order to improve low male participation and mitigate the effect of socio-economic and cultural factors.

## Background

It is estimated that about 25 million HIV-infected people are living sub-Saharan Africa. About 2 million of them are children below the age of 15 years and account for about 90% of all the HIV-infected children worldwide. Over 1700 children become infected with HIV worldwide each day. Over 95% of them get it through mother-to-child transmission (MTCT) [1,2]. In high-income countries, MTCT of HIV has been virtually eliminated through effective voluntary counselling and testing (VCT) for HIV or routine testing of HIV, the use of antiretroviral therapy and the use of safe, affordable and accessible breast-milk substitutes. Since the year 2000, prevention of mother-to-child transmission of HIV (PMTCT) programmes have been initiated in many resource-poor countries as an integrated service in the antenatal care. Nevirapine has been the most commonly used antiretroviral drug in many of the programmes because it is cheap and easy to administer [3,4]. Studies have shown that the utilisation of PMTCT services by the pregnant women is influenced both by factors related to the health system such as accessibility of VCT services, and by individual factors such as fear of disclosure of HIV results, lack of male partner support, fear of domestic violence, abandonment and stigmatization [5-11].

The Uganda National PMTCT programme started as a pilot intervention in the year 2000 and by the end of 2003 it had expanded to cover 38 sites in 56 districts. It was launched in Mbale Regional Referral Hospital in May 2002. In the year 2003, less than 10% of the pregnant women accepted antenatal testing and couple VCT was low [12,13]. Participation of men in the antenatal care of their spouses and couple VCT increase the utilisation of interventions to prevent HIV-1 transmission [14,15]. From July to August 2004, we conducted this study to determine the level of participation of male partners in the PMTCT programme and to identify factors that determine male participation in this programme.

## Methods

Mbale district is situated at the foothills of Mt Elgon in the eastern region of Uganda. It is divided into three counties (rural) and one municipality (urban); namely Bubulo, Bungohko and Manjiya counties and Mbale municipality. In 2003, the district had a population of over 720,000, of which about 90% were rural and the average household-size was 7 people. About 80% of the residents depend on agriculture (subsistence farming) [16]. Its population is predominantly *Bagisu *and the main language is *Lumasaba/Lugishu*. The HIV prevalence rate in 2002 at the Mbale antenatal sentinel site was 5.9% [17].

The study was carried out in Mbale municipality and the surrounding Bungokho County. These were purposively selected to provide a rural and an urban population. The study consisted of two parts. The quantitative part was a cross-sectional survey of men aged 18 years or more, whose spouses were attending antenatal care at Mbale Regional Referral Hospital and resided in the study area. The qualitative part consisted of 8 focus group discussions and 5 in-depth interviews. We calculated the sample size based on the estimation of the proportion of male involvement (defined as men attending antenatal care with their spouses) in the population of 10%, an absolute precision of 3% and a 5% level of significance. We increased the sample size by 10% to cater for anticipated non-response. Hence the total sample size was estimated to be 422 men.

The participants were recruited into the study through their wives. In July and August 2004, all mothers who were coming for their second antenatal visit at Mbale Regional Referral hospital, and resided in the study area, were informed about the study by the midwives. They were requested to provide information concerning their partners' place of work and residential addresses. Subsequently, the men were traced and 388 of them accepted to participate in the study and they were interviewed (participation rate of 92%). The non-response rate was 8% (6 declined, 28 absent). A pre-tested, structured, interviewer-administered questionnaire used to collect the quantitative data consisted of three parts: (1) socio-demographic characteristics (age, marital status, religion, level of education, occupation, place of residence, ownership of a radio, land and livestock, and type of housing), (2) general perceptions and experiences about the PMTCT programme, and (3) perceptions and experiences about antenatal and postnatal care.

**Figure 1:**
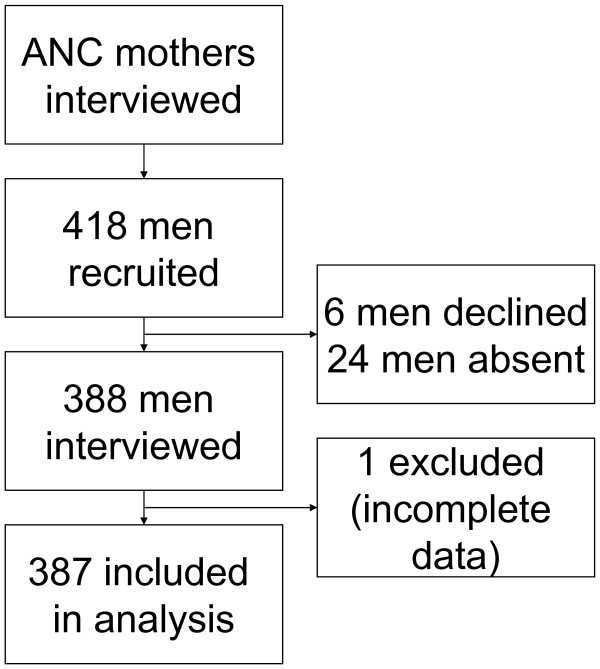
**Study profile of the cross-sectional survey in Mbale, Eastern Uganda**.

The quantitative data was collected by 10 research assistants, entered using EPIDATA http://www.epidata.dk and exported to SPSS version 13 for analysis. Incomplete data of one respondent was not included in the analysis (Figure 1: study profile). The data was summarised and the odds ratios (OR) estimated; and their corresponding 95% confidence intervals (95%CI) were computed. The level of male involvement in PMTCT was determined using an *ad hoc male involvement index*. This index was constructed using six variables with equal weight in the score:

1. The man attends antenatal care with his partner

2. The man knows the partner's antenatal appointment

3. The man discusses antenatal interventions with his partner

4. The man supports his partner's antenatal visits financially

5. The man has taken time to find out what goes on in the antenatal clinic

6. The man has sought permission to use a condom during the current pregnancy

The involvement score for each respondent could range from 0 = no involvement to 6 = involved in all 6 activities. A total score of 4-6 was considered as a 'high' male involvement score and 0-3 as 'low' relative to this particular population.

Bivariate analysis was performed between high male involvement index as the dependent variable and each independent variable. Since the number of variables was small, they were all entered into the binary logistic regression model at a go. Interaction and confounding between the ages of respondents, their place of residence and the significant variables (after bivariate analysis) were checked for.

We also conducted 8 focus group discussions (FGDs) and 5 in-depth interviews. The purpose of the FGDs was to explore and obtain information about the factors hindering male involvement in PMTCT and its influence on the utilisation of the PMTCT programme.

The participants in the FGDs were not selected from male partners of women attending antenatal clinics. Instead participants included men purposively selected from the Aids Information Centre (AIC), the Aids Support Organization (TASO) and the four administrative divisions in Bunghoko county and Mbale municipality.

In each division, two FGDs were held, one for young men aged 18-35 and another for men 36 years and above. The participants were divided into two age groups because we assumed that the young participants would not feel free to discuss or express issues regarding sexuality amidst older participants. The contact person in each of the four administrative divisions assisted in recruiting the participants. Each FGD consisted of 6 to 12 participants. Overall, 76 men participated in the 8 focus group discussions. The FGDs were moderated by an experienced social scientist using a topic guide. Another research assistant (a note taker) recorded the discussions by hand and by tape recorder. Each discussion was conducted in a comfortable place away from distraction with participants seated in a circle. The data was transcribed and summarized into themes.

The topics covered in the focus group discussion included HIV in general - terminology, frequency, causes, prevention, and treatment; HIVin children - terminology, occurrence, causes, prevention, treatment; voluntary counselling and Testing for HIV; stigma and discrimination in HIV; cultural issues; social networks and support for mothers; the health care system; suggestions as to how to involve men in the PMTCT programme.

In addition to the FGDs, in-depth interviews of health care providers were conducted bringing up the following issues about PMTCT: views about voluntary counselling and testing for HIV for pregnant mothers (VCT); testing using the opt-in or opt-out approach; how to increase acceptance of VCT by men; the role of men in promoting VCT in pregnancy, and the acceptability and feasibility of couple VCT. The key informants were also asked about factors influencing male participation in antenatal and PMTCT services in the hospital. Finally, they made suggestions as to how to get male partners more involved in both antenatal care and PMTCT programme.

The health care providers who were interviewed included 3 men and 2 women, namely the Nursing Officer in charge of ANC/PMTCT; the Medical Superintendent of Mbale Hospital; the Reproductive Health Coordinator (Eastern region); the managers of AIC and TASO, Mbale branches respectively. The focus group discussions and the in-depth interviews were conducted mainly in English. However four FGDs were conducted in the local language (Lugishu/Lumasaba). The sessions lasted one to two hours. The data was transcribed, coded using the OpenCode program, version 2.1: June, 2001 and examined for the main themes.

Research and ethical clearances were obtained from Makerere University Faculty of Medicine Research and Ethics Committee, the Uganda National Council of Science and Technology and Mbale District Local Government. Informed consent was obtained from all participants in the study.

## Results

### Socio-demographic characteristics

Of the 387 respondents included in the analysis, 199 (51%) were in the 25-34 age group. The median age was 32 years with inter-quartile range (IQR) of 28-37 years.

Thirty-five percent of the participants were urban, while the rest were rural. The literacy rate was 80% in the urban population and 74% among the rural participants, table 1.

**Table 1:** Socio-demographic characteristics of the 387 respondents who participated in the survey in Mbale, Uganda.

Variable	Respondents N (%)
*Age (years)*	
19-24	49 (13)
25-34	199 (51)
35-44	113 (29)
45 or more	26 (07)
*Place of residence*	
Rural	250 (65)
Urban	137 (35)
*Highest level of education*	
No formal education	25 (07)
Primary	206 (53)
Secondary	127 (33)
Tertiary	29 (07)
*Religion*	
Catholic	69 (18)
Protestant	110 (28)
Muslim	193 (50)
Other	5 (04)
*Marital status*	
Unmarried (single)	11 (03)
Married	376 (97)
*Occupation*	
Builder	26 (07)
Casual labourer	26 (07)
Driver	47 (12)
Farmer	61 (16)
Mechanic	32 (08)
Professional	31 (08)
Trader	143 (37)
Other	21 (05)

The median period of the completed years of schooling by the respondents was 7 years (range: 0-18 years). Fifty percent of the respondents were Muslims. The respondents in the urban areas were more educated compared to those in the rural areas; 8 years compared to 7 years (p-value < 0.001: Mann-Whitney U test). There were more participants whose occupation was farmer in the rural areas than in urban areas, 21% versus 5% (Odds Ratio [OR] = 5.1; 95% Confidence Interval [CI]: 2.3 - 11.5). For the men who participated in the FGDs, their median age was 34 years with an inter-quartile range of 24-44 years.

### Level of male involvement in PMTCT programme

The level of male involvement in PMTCT was assessed using the variables shown in Table 2. Only 18 (4.7%) of the 387 men had attended ANC with their partners, but most of them (377 [97%] out of 387) provided financial support to their spouses to attend ANC. The majority of respondents (236 [61%] out of 387) had not asked their partners whether they (the men) could use condoms during sexual intercourse with them (the women). Only 99 (26%) of the 387 respondents had a high male involvement index.

**Table 2:** Level of involvement of the 387 men in ANC activities.

Item (variable)	Respondents' responses N (%)	
	
	Yes	No
Ever attended ANC with partner	18 (05)	369 (95)
Knows partner's ANC appointments	214 (55)	173 (45)
Provides financial support to partner to attend ANC	377 (97)	10 (03)
Discusses with partner information or interventions given in ANC	114 (30)	273 (70)
Asked partner if he could use a condom	151 (39)	236 (61)
Takes time to find out what goes on in ANC	105 (27)	282 (73)

### Determinants of male involvement in the PMTCT programme and antenatal care

In univariate analysis, the determinants of male involvement in PMTCT programme included: education level, knowing HIV sero-status and having heard about PMTCT.

Men who had had 8 or more years of education were 2 times more likely to get involved in the PMTCT programme than those with less education. Those who knew their HIV sero-status were 4 times more likely to get involved in the PMTCT programme. In addition those who had heard about the PMTCT programme were 2 times more likely to get involved. Those who feared to disclose their HIV status to their spouses were less likely to get involved (Table 3).

**Table 3:** Factors influencing men's involvement in the prevention of mother-to-child transmission of HIV programme (N = 387).

Factor (Variable)	Respondents with high involvement index n/N (%)	Unadjusted Odds Ratio (95% CI)	Adjusted Odds Ratio (95% CI)
*Age (years)*			
19-29	37/146 (25)	1.0	1.0
30+	62/241 (26)	1.0 (0.6-1.6)	1.1 (0.6-1.8)
			
*Education (years)*			
0-7	45/231 (20)	1.0	1.0
8+	54/156 (35)	2.2 (1.4-3.5)**	1.9 (1.1-3.3)*
			
*Residence*			
Rural	65/250 (26)	1.0	1.0
Urban	34/137 (25)	0.9 (0.6-1.5)	0.8 (0.5-1.5)
			
*Religion*			
Muslim	43/193 (22)	1.0	1.0
Christian	56/194 (29)	1.4 (0.9-2.2)	1.1 (0.6-1.9)
			
*Occupation*			
Others	82/309 (26)	1.0	1.0
Driver/Bodaboda cyclists	4/47 (08)	0.3 (0.1-0.7)*	0.3 (0.1-0.9)*
Professional	13/31 (42)	2.0 (0.9-4.3)	1.1 (0.5-2.7)
			
*Fears disclosure of HIV results to wife*			
No	78/252 (31)	1.0	1.0
Yes	14/100 (14)	0.4 (0.2-0.7)**	0.4 (0.2-0.7)**
			
*Knows his HIV sero-status*			
No	78/347 (22)	1.0	1.0
Yes	21/40 (52)	3.8 (2.0-7.4)**	1.9 (0.9-4.4)
			
*Ever heard of PMTCT*			
No	51/257 (20)	1.0	1.0
Yes	48/130 (37)	2.4 (1.5-3.8)**	1.6 (0.9-2.7)

**I**. * is 0.01 ≤ P ≤ 0.05 and ** is P < 0.01. **II**. The variables in the final multivariate logistic regression model were: age, education, place of residence, religion and occupation of the respondents; fears disclosure of HIV sero-status results to his wife, knows his sero-status and ever heard of PMTCT. **III**. The sample size was 387 men (analysed for male involvement index). **IV**. OR, odds ratio; CI, confidence interval; PMTCT, prevention of mother-to-child transmission of HIV; HIV, Human immunodeficiency virus.

On logistic regression, the respondents who had attained secondary education or higher were twice as likely to have a high male involvement index (Table 3). However, those who had fear of disclosure of HIV results to their spouses and men whose occupation was driver were less likely to have a high male involvement index. Age, religion and place of residence of the respondents were not associated with male involvement in PMTCT of HIV (Table 3). Of the 31 professionals, 6 (19.4%) had attended ANC with their wives compared to only 12 (3.4%) of the 356 non-professionals (Table 4). This difference was statistically significant. Men who knew their HIV sero-status or knew their wives' ANC appointments or those willing to go for VCT were more likely to attend ANC with their wives (Table 4).

**Table 4:** Factors influencing men's participation in antenatal care with their partners in Mbale district, Uganda (N = 387)

Factor (Variable)	Not Attended ANC n = 369	Attended ANC n = 18	Unadjusted OR (95% CI)	Adjusted OR (95% CI)
*Age (years)*				
19-29	142	4	1.0	
30+	227	14	2.2 (0.7-6.8)	
*Residence*				
Rural	236	14	1.0	
Urban	133	4	0.5 (0.2-1.6)	
*Education (years)*				
0-7	222	9	1.0	
8+	147	9	1.5 (0.6-3.9)	
*Religion*				
Other	188	5	1.0	
Christian	181	13	2.7 (0.9-7.7)	
*Occupation*				
Other	344	12	1.0	1.0
Professional	25	6	6.9 (2.4-20)**	5.4 (1.7-17)**
*Knows his HIV sero-status*				
No	335	12	1.0	
Yes	34	6	4.9 (1.7-14)**	
*Bothers to know what goes on in ANC*				
No	276	6	1.0	1.0
Yes	93	12	5.2 (1.9-14)**	3.1 (1.2-9.2)*
*Knows his wife's ANC appointments*				
No	172	1	1.0	1.0
Yes	197	17	14.8 (2 - 110)**	8.6 (1.0-72)*
*Discussed with wife ANC interventions*				
No	267	6	1.0	
Yes	102	12	5.2 (2.0-14)**	
*Willing to go for HIV test*				
No	122	1	1.0	
Yes	247	17	8.4 (1.1-63)*	
*Ever heard of PMTCT*				
No	249	8	1.0	
Yes	120	10	2.6 (1.0-6.7)*	
*Would go with wife for VCT*				
No	205	4 (2)	1.0	1.0
Yes	164	14 (8)	4.4 (1.4-13)	3.6 (1.1-12)

Notes: **I**. * is 0.01 ≤ P ≤ 0.05 and ** is P < 0.01. **II**. All variables in the above table were entered in the final multivariate logistic regression model. **III**. OR, odds ratio; CI, confidence interval; PMTCT, prevention of mother-to-child transmission of HIV; HIV, Human immunodeficiency virus; ANC, antenatal care.

### Barriers to male participation in the PMTCT programme

Factors hindering men's participation in the PMTCT programme that were identified in the focus group discussions were related to the health system, to socio-economic status and to culture.

#### Health system factors

Several factors related to the health system were identified as barriers to male participation in the ANC. The first major factor consistently identified by all the focus groups was rudeness and rough handling of the pregnant women by the health-workers in the antenatal clinics, as reflected in the following men's responses:

*"Medical personnel handling pregnant mothers are very rough especially when it comes to examination of the abdomen"*, said a respondent from Bongokho sub-county. Another one from the AIC focus group said, "*During check-ups midwives over-press the pregnant mother's abdomen. We are fed up with the female health-workers. These midwives are very rude to the mothers. They are too harsh and abuse the pregnant women"*.

The second factor reported by the respondents was that in some instances the health-workers do not allow them to enter the antenatal clinics with their pregnant women.

A third factor cited as one of the barriers to male participation was the charging of un-official user-fees. Lack of adequate space in the antenatal clinics was cited as a fourth factor. One respondent said: *"The clinics are congested. There is not enough space to accommodate the women and their husbands. Men will not feel comfortable sitting with women who are strangers to them. They will rather wait outside and if the procedures take long they will leave"*. The last factor was the geographical distance to the services, one respondent said: *"The antenatal clinics are far from the local people"*

#### Socio-economic factors

The second area mentioned as an obstacle to male participation was socio-economic. Several of the men reported that due to socio-economic difficulties, they did not have time to attend ANC with their partners as demonstrated by the following quotations: *"I am busy trying to make ends meet. I don't have time to go with her to the antenatal clinic. I don't have enough money for transport for two people*", said a respondent from a rural village. *"We have to struggle to look for money to provide for our families"*, said a respondent from Bongokho FGD.

#### Cultural factors

The third hindrance to male participation was cultural beliefs. A negative attitude of some men was common, as demonstrated by the AIC focus group discussion: *"I believe it is not good to follow your wife to the antenatal clinic. Even though she exposed her privacy to you at home but when you reach the antenatal clinic it is different. So it is better she goes alone"*. Another one said, *"If I accompanied my wife to hospital every time she goes for her antenatal check up, my friends would think I am a weakling. They would laugh at me"*. One of the key informants said: "*Because of cultural beliefs, most men do not like to accompany their wives to the antenatal clinics. Men who accompany their wives to ANC are perceived to be weaklings by their peers"*.

### Respondents' suggestions for improving male involvement in ANC/PMTCT activities

The suggestions as to how improvements could be made came from mainly health care providers (key informants). However the participants (males) in the FGDs were also emphatic on their recommendations as to how to improve the situation and their suggestions included the following:

1. Sensitize men about ANC and PMTCT, and their benefits

2. Conduct refresher courses for midwives and nurses

3. Men should be invited by staff to attend ANC using the ANC cards of their wives

4. Government should bring services closer to the people

5. Welfare of the staff should be improved

6. More staff be recruited into the health service

Specifically, the respondents suggested that retraining of the health-care providers should include customer-care skills. In addition they suggested provision of better remuneration to the health workers and building more health units by the government closer to the local people where antenatal care could be offered.

Some respondents suggested that midwives should write on the antenatal cards informing the men to come with their wives on subsequent ANC visits. A respondent from the Bungokho focus group said: *"Men should be identified from the communities, trained on PMTCT and then sent back to the communities to inform and mobilize other men. Health workers should use all fora to inform men about PMTCT; the churches and mosques are good entry points"*. Another one said:

"Midwives should be given refresher courses because they seem to be losing direction. Nurses and midwives should become friendlier to the mothers in ANC than they are at the moment"

## Discussion

In this study, conducted to establish determinants of male involvement in the PMTCT programme in Eastern Uganda, we found that only 1 in 4 male partners were involved in the PMTCT programme. This level of involvement is low but higher than what is reported from other studies from East Africa [18]. For example one study from Mulago Hospital in Kampala, Uganda, showed that male participation in the PMTCT activities was low (16%) [14,18]. Similarly, a study conducted at a Nairobi antenatal clinic, Kenya revealed that male partner participation in antenatal VCT with their spouses was low (15%) [14]. The difference between our findings and these other studies could be attributed to the different methods used.

In this study we have found a number of factors associated with male participation in the PMTCT programme. These included heath system, socio-economic and cultural factors. First the structural set up of the ANC clinics is not male user-friendly. Furthermore, our findings from the qualitative data have indicated that the health-providers in the ANC clinics were perceived by the men not to be client-friendly. Similar observations have also been made by others. For example focus group participants in Kenya mentioned rude health care staff as a hindrance to participation in PMTCT services [19].

Other factors hindering male involvement in the PMTCT programme were socio-economic. Although the majority of the men expressed willingness to go for HIV counselling and testing, they said that they either lacked time or money to facilitate their involvement in the ANC/PMTCT activities. Similar constraints have been found in Dodoma, Tanzania [20]. In addition, the level of education and occupation of the respondents influenced male participation in the PMTCT programme. Similar studies in Uganda and elsewhere have found that education level is an important determinant of participation in PMTCT services [21,22].

Increased access to information, knowledge and awareness facilitates good choices. In our study men who had heard about PMTCT programme were 2 times more likely to get involved in PMTCT activities than those who had not. This is consistent with results of a similar study in Mambwe district in Zambia [23].

In addition, another study from Chipata in Zambia revealed that males were not fully participating in PMTCT programmes and reasons given were lack of information and lack of a direct link between PMTCT staff and males [24].

Cultural factors were also found to be hindering male involvement in the PMTCT programme in Mbale. For example antenatal care was perceived a women's affair.

As has been shown in other studies [25], it is conventional in many African cultures for men not to accompany their partners to antenatal and postnatal care consultations as pregnancy and child birth are regarded as a women's affair [26,27].

One of the strengths of this study is that we interviewed men and not their spouses. The information collected in this study is likely to reflect the men's views better than information obtained from women. The other strength is that we used both quantitative and qualitative methods during data collection. The qualitative findings assisted in explaining the findings from the quantitative part of the study.

There are some potential weaknesses, though, in the methodology. First, the recruitment of respondents through their spouses in the ANC in a hospital setting could have introduced selection bias. In this study, the level of non-response was modest (7%), unlikely to have biased our estimates in any major way. Second, it is possible that husbands of the women attending ANC were different from the partners of the women who did not attend ANC. However, since over 90% of women in Uganda attend at least one ANC visit [28], the potential selection bias is limited.

Third, our male involvement index has not been used before and its validity and reliability have not been established in this environment. To our knowledge, there exists no established instrument to assess male involvement. We still believe this index is likely to divide the men in the two groups of high and low involvement relative to this population, but not in absolute terms as even the 'high involvement' group represents a modest involvement in absolute terms.

PMTCT programmes have been started at different times in the different African countries. The programmes with the longest history demonstrate many similarities in their development from start-up to a mature programme. Men's involvement and understanding of any PMTCT programme is likely to develop along with the maturation of the programme. This study was conducted in the early days of the programme and the study results may in part reflect this. In a follow-up study it would be interesting to assess how much has changed since 2003.

## Conclusions and recommendations

The level of male involvement in PMTCT programme in Mbale was low in 2003. Several factors appear to contribute including health system factors such as: health workers' behaviour and unfriendly environment and clinics designed for women. Socio-economic factors such as costs of transport, education level, occupation and culture also contribute.

Improvements in the health care system and community sensitization of men about the benefits of antenatal care and the PMTCT programme are essential for mitigating the effect of socio-economic and cultural factors.

## Competing interests

The authors declare that they have no competing interests.

## Authors' contributions

RB participated in the conception, design, and implementation of the study, statistical analysis and interpretation of the data, and the drafting of the manuscript. JKT participated in the conception, design, and supervision of the study, statistical analysis and interpretation of the data, and the drafting of the manuscript. NS participated in the conception, design and implementation of the study, and the drafting of the manuscript. TT participated in the conception and design of the study, statistical analysis and interpretation of the data, and the drafting of the manuscript.
